# A Novel Message-Preserving Scheme with Format-Preserving Encryption for Connected Cars in Multi-Access Edge Computing

**DOI:** 10.3390/s19183869

**Published:** 2019-09-07

**Authors:** Insu Oh, Taeeun Kim, Kangbin Yim, Sun-Young Lee

**Affiliations:** Department of Information Security Engineering, Soonchunhyang University, Asan 31538, Korea (I.O.) (T.K.) (K.Y.)

**Keywords:** controller area network (CAN), format-preserving encryption (FPE), connected car, preserving scheme, multi-access edge computing (MEC)

## Abstract

In connected cars with various electronic control unit (ECU) modules, Ethernet is used to communicate data received by the sensor in real time, but it is partially used alongside a controller area network (CAN) due to the cost. There are security threats in the CAN, such as replay attacks and denial-of-service attacks, which can disrupt the driver or cause serious damage, such as a car accident through malicious manipulation. Although several secure protocols for protecting CAN messages have been proposed, they carry limitations, such as combining additional elements for security or modifying CAN messages with a limited length. Therefore, in this paper, we propose a method for encrypting the data frame, including real data in the CAN message structure, using format-preserving encryption (FPE), which ensures that the plaintext and ciphertext have the same format and length. In this way, block ciphers such as AES-128 must be divided into two or three blocks, but FPE can be processed simultaneously by encrypting them according to the CAN message format, thus providing better security against denial-of-service attacks. Based on the 150 ms CAN message, a normal message was received from a malicious message injection of 180 ms or more for AES-128 and a malicious message injection of 100 ms or more for FPE. Finally, based on the proposed scheme, a CAN transmission environment is constructed for analyzing the encryption/decryption rate and the process of transmitting and processing the encrypted message for connected cars in multi-access edge computing (MEC). This scheme is compared with other algorithms to verify that it can be used in a real environment.

## 1. Introduction

Early cars evolved into connected cars to provide transportation for people or goods, but cars were interconnected and controlled by various devices. In addition, many electronic control units (ECUs) control the vehicle by connecting them with various sensors, communication systems, and driving control systems. ECU is a general term for various electronic control devices of a vehicle. In addition to engine control, ECU is used in various automobile modules, such as that of transmission, the airbag, and tire pressure. Recent advances in automotive technology have created convenient functions, such as active cruise control (ACC) and lane departure avoidance, which facilitate safe driving. Various electronic control devices and sensors are mounted inside the automobile to implement these convenience functions. Modules in the car exchange necessary data during operation and communicate through an internal network. FlexRay, media-oriented systems transport (MOST), Ethernet, the controller area network (CAN), and other networks are used to process vehicle information, such as acceleration, braking, and button operation. In recent years, ECUs have been connected via Ethernet to provide high-capacity and high-speed processing in preparation for the greater use of autonomous vehicles. Most cars use the controller area network (CAN) as an internal network due to its cost [1,2].

The CAN is the most popular network used to classify messages according to their IDs without a host and exchange necessary data between ECUs. However, the CAN is not safe from replay attacks because it does not authenticate their original source. The reason for this is that a CAN is composed of a bus network and data is broadcasted to ECUs at all nodes, and packets are identified through their IDs, making a CAN vulnerable to replay attacks. In addition, there is a possibility of denial-of-service attacks, which cause ECU processing delays by generating messages with a high priority because the message is processed according to priority based on the ID of the message. In addition, fuzzing the unspecified messages can cause the car to malfunction, resulting in serious consequences. The last CAN message can be used as an important data source for car accident analysis in the future.

As such, an attacker can manipulate a message and send it or expose it by sniffing. Therefore, we need a way to protect CAN messages. To protect such important CAN messages, authentication methods such as the message authentication code (MAC) are used for message authentication or only messages in a certain period are processed as valid messages through synchronization. However, it is difficult to guarantee the security of messages against a denial-of-service attack as using an extra message or data to improve security decreases the processing speed of the system for all additional messages.

Our proposed method does not require any additional messages for message encryption in a CAN. Instead, message confidentiality is ensured by encrypting 8 bytes using format-preserving encryption (FPE) in the CAN frame. In addition, it is possible to prevent a denial-of-service attack by generating a single message, even though messages are encrypted. Finally, the applicability in a real CAN environment can be verified by comparing and analyzing the security strength, encryption speed, and performance of the FPE algorithm with a CAN transmission environment. By applying our proposed method, we can prevent confidentiality and re-play attacks through sending a single CAN message to the connected car in multi-access edge computing [3].

The paper is organized as follows. Section 2 describes CAN networks, security threats to CAN, and existing message protection measures. Section 3 presents the proposed scheme for encrypting CAN messages with FPE. Some comparative simulation experiments and their results are presented and discussed in Section 4. Section 5 verifies the applicability to real CAN networks, which is followed by concluding remarks and future directions presented in Section 6.

## 2. Related Work

### 2.1. CAN

**Figure 1:**
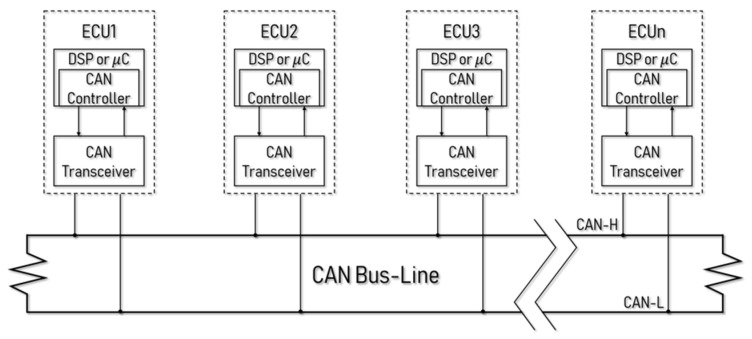
The controller area network (CAN) structures.

CAN is the most widely used automobile network and is the communication standard between controllers without a host. Existing vehicles contain a small number of modules, so device-to-device communication between ECUs via a universal asynchronous receiver/transmitter (UART) has been used, but the number of connected ECUs increases due to the additional cost required for a connection. Therefore, the CAN emerged to address the cost, speed, and performance problems for connecting all the modules. For a car manufacturer, the CAN bus is divided into three types of function, and the structure is shown in Figure 1. Each ECU contains a microcontroller unit (MCU) and has a controller to handle CAN messages and a transceiver to manage received messages [4,5].

A CAN is a bus-shaped peer-to-peer network. Because all ECU nodes share the message with two CAN high (CAN-H) and CAN low (CAN-L) lines, the configuration is simple and economical. This also provides a stable network with less interference owing to the use of twisted pair cabling. All messages have priority, and if two nodes send messages simultaneously, high priority messages are sent first. Carrier sense multiple access with collision avoidance (CSMA/CA) is used to learn that the other host is transmitting data and waits for a random amount of time, and the arbitration on message priority (AMP) policy is used to determine the relative importance of a message [4,5].

The structure of a CAN data frame is divided into two versions, depending on the length of the identifier. A standard identifier is 11 bits and the extended version is 29 bits. The data frame structure is divided into five parts: arbitration, control, data, check, and acknowledge (ACK). Table 1 shows the CAN data frame structure [4].

### 2.2. Security Threats in a CAN

Security threats of automotive ECUs include internal and external attacks. Security threats through CANs are attacks that are made by physically accessing the internal CAN bus, because the CAN bus broadcasts messages and these messages are sent to all nodes. Therefore, an attacker that can physically access the CAN bus is likely to steal messages to achieve various security threats, including message insertion, replay attacks, and denial-of-service attacks.

The structure of the CAN frame is defined as the standard; nevertheless, it is modified slightly by each manufacturer and car model. If the attacker collects CAN messages through eavesdropping and interprets the meaning, the attacker can implement a tool to monitor each message and send a message to induce a malicious specific action. In this paper, we propose a CAN packet analysis tool called CarShark, which can easily access and interpret the CAN data. This can modify the data frame to manipulate the revolutions per time (RPM) or speed or cause a warning light to be turned on for the cluster, which can cause serious accidents during driving [6,7].

The CAN data frame verifies error in the data through the cycle redundancy check (CRC) bits, but it does not distinguish normal and abnormal frames. Therefore, if the attacker grasps the ID of the ECU by eavesdropping, determines the attack target, generates a fake frame, and continuously transmits to the ECU, the ECU will accept the fake frame and process the message [8].

In bus communication, each node is connected by a single cable. A collision occurs if two different nodes transmit data simultaneously. At this time, the CAN arbitrates based on the priority of the message. A high value of the identifier in the arbitration field of the CAN message indicates that the message has high priority. As a result, if an attacker maliciously sends a high-priority message to the CAN bus, a normal CAN message can collide with the attacker’s message and the inserted message can be selected in terms of priority. In this way, a denial-of-service attack will succeed if many high-priority frames are generated and transmitted to ECUs. Therefore, the ECU is occupied with resources for processing the message, and a delay occurs in processing the normal frame [9,10].

### 2.3. CAN Message Protection

In the case where a CAN message has a bus-type structure, a representative example of a denial-of-service attack is an attack where a message is inserted to cause an intended malicious operation and paralyze a bus. Because the attack targets buses, security threats occur when unauthorized messages are injected into the bus. In recent studies, several methods have been proposed to prevent the attack using message authentication, by periodically monitoring messages, and by encrypting and decrypting messages using a key that is shared in advance, which can improve security in a CAN.

In the case of TESLA [11], a one-way keychain is generated using time synchronization, and keys are sequentially allocated and used for specific time intervals. This key is used to generate a MAC value and verify it to authenticate the message.

In the case of centralized authentication system in CAN (CaCAN) [12], hash-based message authentication (HMAC) is used to add a digest to a few bits after the normal message as an authentication factor. Using this, we can continuously verify the HMAC through the monitoring node, authenticate the message, and add tested nodes to exchange secret values between ECUs.

In the case of message authentication protocol on the CAN bus (CanAuth) [13], HMAC is used in the same way, but the structure of the key configuration frame for sharing a key in advance and the structure of the message used for authentication of the normal message are different from the frame for transmitting general data.

In the case of a lightweight broadcast authentication protocol for CAN (LiBrA-CAN) [14], the key is distributed to each node and verified at the center. It uses mixed message authentication codes (M-MAC) that employs MAC multiple times. M-MAC, which is a linear hybrid MAC that fuses multiple authentication tags in a single tag, is used to generate an authentication tag for the key using the key arrangement. The authentication frame and the message frame of the key are authenticated through the authentication tag. Therefore, the above four protocols are safe from denial-of-service attacks, but they do not provide confidentiality.

In the case of a lightweight can authentication protocol (LCAP) [15], a magic number chain is generated through a one-way hash function and a random number generator, and mutual authentication is performed through a handshake. It also protects the message with the RC4 encryption algorithm using the session key. Therefore, it is safe from denial-of-service attacks and provides confidentiality Nevertheless, additional messages must be sent for channel configuration and synchronization.

In the case of Vector [16], a random ID value is generated at the beginning of communication and transmitted to the sender, thereby creating a kind of session that processes only messages with matching IDs. The ID key is periodically increased to prevent replay attacks. In addition, in the CAN with flexible data (CAN-FD) environment, messages are encrypted with AES-128 to satisfy or provide confidentiality of the message, but they are not safe from a denial-of-service attack.

As shown in the above Table 2, most prior studies have focused on preventing a message injection attack and denial-of-service attack using message authentication. However, including new embedded hardware in an existing CAN is difficult owing to the additional elements that must interface with the ECU. Additionally, because a larger number of messages are transmitted/received than in the case of processing existing CAN messages, there is a possibility of network overload in a bus network, where existing messages cannot be processed as the number of messages to be processed by each ECU increases. Therefore, in this paper, we propose a new scheme that can satisfy the confidentiality of messages based on FPE. This scheme complies with existing CAN standards and can be included within a CAN.

### 2.4. Format-Preserving Encryption (FPE)

For the security of personal information, the encryption of personal information is mandatory in a database. However, applying a block cipher such as advanced encryption standard (AES) is problematic as the storage space is wasted because the length is fixed. Personal information is stored in a certain format using fixed-length fields for efficiently using storage space and improving the retrieval performance. Therefore, if an existing block cipher is used for personal information in a database, the form changes and the database must be redesigned. FPE has been proposed to solve this problem. Because FPE has the feature that the length and format of the encrypted data do not change, it has an advantage in that it can be used in an environment where the data length and format must be preserved [17].

**Figure 2:**
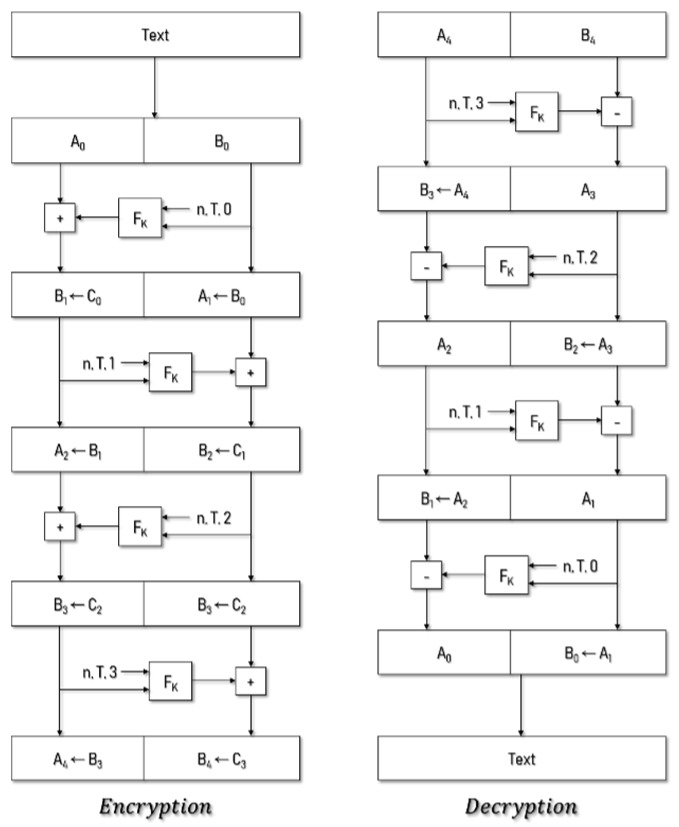
Encryption and decryption structure of format-preserving encryption (FPE) [17].

The cryptographic domain of FPE can be vulnerable to codebook attacks because it can be very small in size. To counter this, tweak (T) is an additional piece of information that is used as an input in FPE with certain types of data, and it need not be kept secret. Because the ciphertext is different every time $T_{n}$ is changed for the same plain text, the problem where plaintext can be deduced from ciphertext is resolved, even if the length and format of the plaintext and ciphertext match [17]. The two standards for FPE (FF1 and FF3-1) were developed by the National Institute of Standards and Technology (NIST). FPE has a Feistel structure, as shown in Figure 2. The FF1 algorithm was used in this study.

## 3. Proposed Scheme

The proposed CAN message protection scheme is presented in this section. The proposed scheme consists of encryption/decryption processes using key generation/distribution and FPE. The key is newly generated every time the engine is started and transmitted to each ECU from the gateway ECU. Keys are broadcasted through a bus in the gateway ECU and a message is received from another ECU, which decrypts the message.

Previous studies have verified the integrity of messages using message authentication, but confidentiality against eavesdropping could not be guaranteed. Therefore, it is necessary to apply encryption to ensure message confidentiality, and a symmetric key-based encryption algorithm must be used because encryption/decryption with a public key-based encryption algorithm takes a significant amount of time. Although triple data encryption algorithm (TDEA) and AES-128 are typical block ciphers, the block sizes are 64 and 128 bits, respectively.

**Figure 3:**
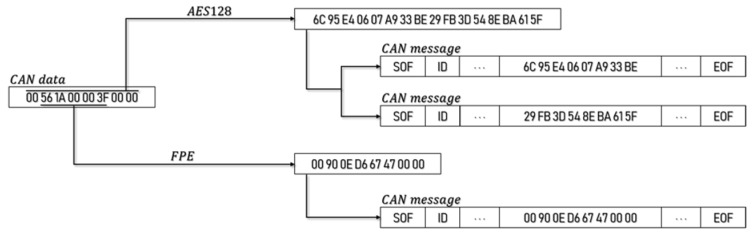
AES-128 and format-preserving encryption (FPE) in a controller area network (CAN) message.

**Figure 4:**
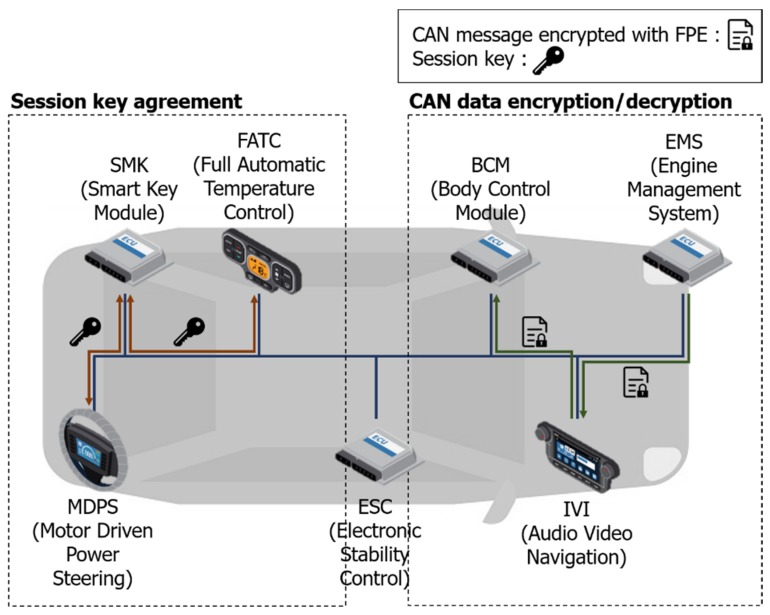
Key generation and distribution and controller area network (CAN) message encryption using the format-preserving encryption (FPE) scheme.

In the case of the CAN frame data field, TDEA can be used with 64 bits, but it cannot guarantee safety compared with AES. In the case of AES-128, two messages must be encrypted and transmitted in total [18]. As shown in Figure 3, two messages must be transmitted, and the recipient must decrypt both packets to obtain a normal message. However, the FPE algorithm is more efficient because it can be transmitted as a single CAN message, due to the form and length being preserved, even if the data is encrypted. The proposed scheme is shown in Figure 4.

The overall process of the proposed scheme is as shown in Figure 4. The scheme includes generating and distributing an encryption key using FPE. We propose a method to effectively improve the security and protect the CAN data frame, without modifying the message structure.

### 3.1. Key Configuration and Distribution

ECUs hold master keys, *K*, such as serial numbers, and hold one seed value of $T_{n}$, which is the same value set by the vehicle manufacturer. The session keys (*SK*) for encryption are generated by each other based on the ID of a message generated by each ECU when the driver starts the engine for the first time. $T_{n}$ is used in FPE, and it must be updated when the message is sent or received. The current $T_{n}$ is used to generate the next $T_{n}$ with a hash algorithm. The method used by ECU_1 and ECU_2 to generate a session key with a master key and the method for updating $T_{n}$ are as Table 3 and Algorithm 1.

**Algorithm 1** Session Key Agreement.1: Pre-shared *K*, $T_{s}$.2: Automotive VCC on.3: ECU_1 generate *na*.4: ECU_1 send ECU_2 : *na*
**then**
$T_{n}=H\left(T_{s}\right)$.5: **If** Id **in** ECU_2 Id filter:  ECU_2 received *na*
**then**
$T_{n}=H\left(T_{s}\right)$.   ECU_2 generate *nb*.   $C=fpeE_{K}^{T_{n}}\left(na⊕nb\right)$.   ECU_2 send ECU_1 : *C*
**then**
$T_{n+1}=H\left(T_{n}\right)$.   $SK=fpeE_{K}^{T_{n}}\left(na∥nb\right)$.6: **If** Id **in** ECU_1 Id filter:ECU_1 received *C*
**then**
$T_{n+1}=H\left(T_{n}\right)$.$nb=fpeD_{K}^{T_{n}}\left(C\right)⊕na$.$SK=fpeE_{K}^{T_{n+1}}\left(na∥nb\right)$.$C=fpeE_{SK}^{T_{n+1}}\left(nb\right)$.      ECU_1 send ECU_2 : *C*
**then**
$T_{n+2}=H\left(T_{n+1}\right)$.7: **If** Id **in** ECU_2 Id filter:ECU_2 received *C*
**then**
$T_{n+2}=H\left(T_{n+1}\right)$.  ${nb}^{\prime }=fpeD_{SK}^{T_{n+1}}\left(C\right)$.**If***nb* == *nb*′:8:     Success generate *SK*.**else**:Goto Row 2.

When ECU_1 generates *na* and transmits it to ECU_2, ECU_2 generates *nb* modular arithmetic with *na* received from ECU1. Additionally, the value obtained by the modular arithmetic is encrypted using the master key and the current $T_{n}$. ECU_2 subsequently transmits the ciphertext to ECU_1 and generates the *SK*, and ECU_1 decrypts the ciphertext received from ECU_2. The previous tweak value is used for decryption. ECU_2 generates an *SK* based on the decrypted value, encrypts *nb* with the generated *SK*, and transmits it to ECU_2. Finally, ECU_2 compares the received ciphertext with *nb* in the decrypted ciphertext received by ECU_1 and confirms that the distribution of the *SK* is performed normally.

### 3.2. CAN Encryption/Decryption with FPE

**Figure 5:**
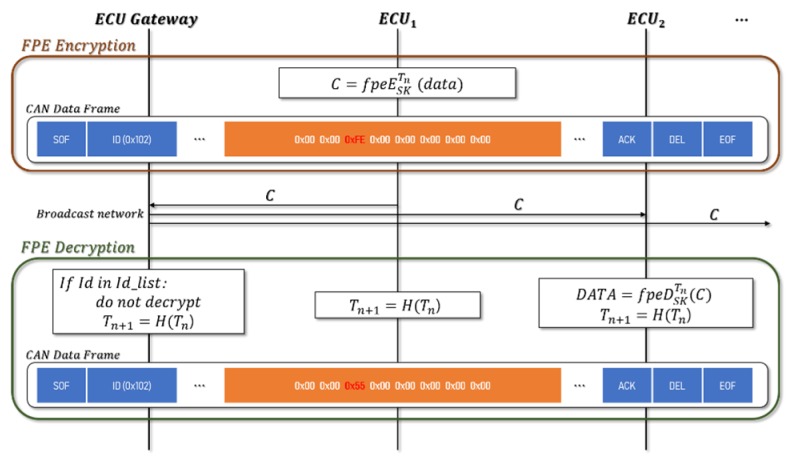
Encryption and Decryption using the format-preserving encryption (FPE).

The *SK* and $T_{n}$ generated and distributed by each ECU are used to encrypt the FPE. When ECU_1 tries to transmit a CAN message, the data field (8 bytes) is encrypted with FPE, and $T_{n}$ is renewed after transmission. Upon receipt of the encrypted CAN message, ECU_2 updates Tn and decodes it using the old $T_{n}$ value to process the message as a normal message. In the case where ECU_3 receives a message but does not decrypt it, it only updates the $T_{n}$ value and prepares it for the next ciphertext. The CAN message has a different data field, depending on the data length. Figure 5 shows an example of the encryption of a message whose data length is 1 byte using FPE.

**Algorithm 2** Controller area network (CAN) data field encryption/decryption1: Pre-shared $T_{n}$, *SK*.2: $C=fpeE_{SK}^{Tn}\left(data\right)$.3: ECU_1 send ECU_2 : *C*
**then**
$T_{n+1}=H\left(T_{n}\right)$.4: **If** Id **in** ECU_2 Id filter:  ECU_2 received *C*
**then**
$T_{n+1}=H\left(T_{n}\right)$.*data*$=fpeE_{SK}^{Tn}\left(C\right)$.
**else**
     $T_{n+1}=H\left(T_{n}\right)$.

Algorithm 2 presents CAN encryption/decryption using FPE. Encrypting portions of the data rather than the entire CAN frame is more efficient than other encryption methods. In addition, the encrypted CAN frame can only confirm the contents of the frame by the ECU_2 possessing the key. This ensures confidentiality because it is difficult to deduce an action or function from the corresponding packet. The detailed procedure of FPE is as Table 4, Algorithms 3 and 4.

**Algorithm 3** FPE_FF1_Encrypt (*K*, $T_{n}$, *CANdata*)**Input**: *K*, $T_{n}$, *CANdata***Output**: Encrypted *CANdata***Steps**:1.  Let *u* = ⌊n/2⌋; v = *n-u*.2.  Let *A* = *CANdata*[1..u]; B = *CANdata*[u+1..n].3.  Let *b* = ⌈⌈v⋅LOG(16)⌉/8⌉.4.  Let *d* = 4⌈b/4⌉+4.5.  Let *P* = ${\left[1\right]}^{1}∥{\left[2\right]}^{1}∥{\left[1\right]}^{1}∥{\left[16\right]}^{3}∥{\left[10\right]}^{1}∥{\left[u\;mod\;256\right]}^{1}∥{\left[n\right]}^{4}∥{\left[t\right]}^{4}$.6.  For *i* from 0 to 9:      i.  Let *Q* = $Tn∥{\left[0\right]}^{\left(-t-b-1\right)\;mod\;16}∥{\left[i\right]}^{1}∥{\left[BTN\left(B\right)\right]}^{b}$.     ii.  Let *R* = BEF(P ∥ Q).    iii.  Let *S* be the first d bytes of the following string of ⌈d/16⌉ blocks:        $R∥BC_{K}\left(R⊕{\left[1\right]}^{16}\right)∥BC_{K}\left(R⊕{\left[2\right]}^{16}\right)\ldots BC_{K}\left(R⊕{\left[\lceil d/16\rceil -1\right]}^{16}\right).$    iv.  Let y = BTN(S).     v.  If *i* is even, let *m* = *u*; else, let *m =* v.    vi.  Let *c* = $\left(NTR\left(A\right)+y\right)\;mod\;16^{m}.\;$   vii.  Let *C* = $ST_{m}\left(c\right)$.     viii.  Let *A = B*, let *B = C*7.   Return A ∥
*B*

**Algorithm 4** FPE_FF1_Decrypt (*K*, $T_{n}$, Encrypted *CANdata*)**Input**: *K*, $T_{n}$, Encrypted *CANdata***Output**: *CANdata***Steps**:1.  Let *u* = ⌊n/2⌋; v = *n-u*.2.  Let *A* = Encrypted *CANdata*[1..u]; B = Encrypted *CANdata*[u+1..n].3.  Let *b* = ⌈⌈v⋅LOG(16)⌉/8⌉.4.  Let *d* = 4⌈b/4⌉+4.5.  Let *P* = ${\left[1\right]}^{1}∥{\left[2\right]}^{1}∥{\left[1\right]}^{1}∥{\left[16\right]}^{3}∥{\left[10\right]}^{1}∥{\left[u\;mod\;256\right]}^{1}∥{\left[n\right]}^{4}∥{\left[t\right]}^{4}$.6.  For *i* from 9 to 0:      i.  Let *Q* = $T∥{\left[0\right]}^{\left(-t-b-1\right)\;mod\;16}∥{\left[i\right]}^{1}∥{\left[BTN\left(A\right)\right]}^{b}$.     ii.  Let *R* = *BEF*(P ∥ Q).    iii.  Let *S* be the first d bytes of the following string of ⌈d/16⌉ blocks:        $R∥BC_{K}\left(R⊕{\left[1\right]}^{16}\right)∥BC_{K}\left(R⊕{\left[2\right]}^{16}\right)\ldots BC_{K}\left(R⊕{\left[\lceil d/16\rceil -1\right]}^{16}\right).$    iv.  Let y = BTN(S).     v.  If *i* is even, let *m* = *u*; else, let *m =* v.    vi.  Let *c* = $\left(NTR\left(B\right)-y\right)\;mod\;16^{m}$.   vii.  Let *C* = $ST_{m}\left(c\right)$.     viii.  Let *B = A*, let *A = C*7.   Return A ∥
*B*

CANdata, which is the data field value in the CAN message, and Tn, which is synchronized with each ECU, are encrypted/decrypted using the key K, which is divided into input values. CANdata is divided into two parts: *A* and *B*. *A* = *B* when the length of the plaintext *n* is an even number, the length of *A* is equal to the length of *B*, and *A* = *B* − 1 when *n* is odd. The variables *b*, *d*, and *P* are defined for dividing the CANdata into two parts and then performing the round of the Feistel. *b* and *d* are the values for maintaining the block length in the algorithm and *P* and *Q* are the values for setting the initial block in block cipher encryption function (BEF). LOG refers to the base 2 logarithm, *b* refers to the number of bytes of *B* to be defined within the face’s round, and *P* is used as the initial block of the BEF. After dividing the plaintext and defining the basic variables, 10 Feistel chipper rounds are performed, yielding the binary string *Q* through Tn, the number of rounds *i*, and the variable *b* synchronized in the network. *Q* and *P* are input to the BEF function, and the output *R* is expanded or reduced to *S* by a string of *d* bytes. *S* is a string of numbers *y* and is added to mod 16^*m* by adding *y* and *A* to each round, and its output value is *c*. Finally, *c* is transformed into a string *C*, so *B* = *A* and *C* = *B* for the next round. The concatenated output values *A* and *B* are obtained by repeating this process 10 times, yielding the ciphertext in the data field of the CAN message [17].

The FF1 decryption process is similar to the encryption process, but there is a slight difference in rounding. First, the round number is inverted from 9 to 0 when proceeding with the rounding function. In addition, the roles of *A* and *B* change, and the modulo addition operation during encryption changes to modulo subtraction to decode the data field in the CAN message.

## 4. Assessment

To verify whether the proposed scheme can be used in a real CAN, we compared the encryption/decryption rate of FPE, which is a symmetric key cipher algorithm in an ECU environment, with other types of symmetric key cipher algorithms. We compared and evaluated the transmission rate to confirm the possibility of a denial-of-service attack. We used NIST 800-22, which tests the randomness of the binary to measure the security strength of the encrypted data and whether the attacker has a possible prediction.

### 4.1. Security Assessment

**Figure 6:**
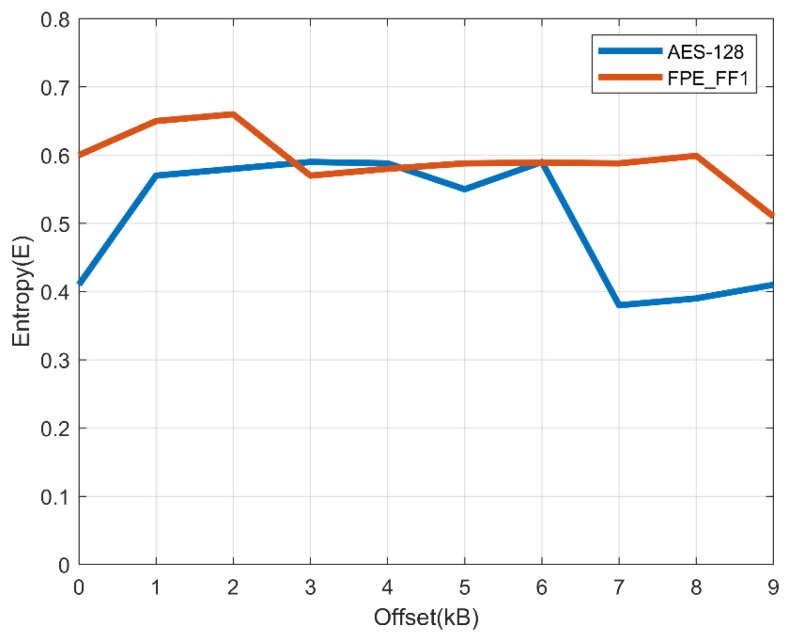
Entropy of the AES-128 and format-preserving encryption (FPE)_FF1 algorithms.

To measure the strength of cryptographic algorithms, the randomness of cryptograms produced with algorithms should be checked. The entropy of the ciphertext based on the AES-128 and FPE algorithms is shown in the following Figure 6.

The NIST 800-22 standard defines 15 test suites to test random number generators and statistically analyze the randomness of ciphertext binaries. A statistical package can be used to test the randomness of binary sequences generated by hardware or software-based cryptographic random number generators or pseudo-random number generators. This test focuses on the various types of determinism that can exist in a sequence [19].

In total, 10,000 random key sets with the same length and plaintext were generated and encrypted, and the generated ciphertext was extracted as binary data. Furthermore, 15 randomness tests were conducted with 10,000 ciphertext binaries using the open source Python software provided by NIST [19]. The results are shown in Table 5 below.

In a statistical hypothesis test, the *p*-value is the lowest probability when the null hypothesis is true for a given statistical model. Statistical summaries will be greater than or equal to. The resulting binary is deemed random when *p* ≥ 0.01. As with the AES-128 algorithm, FPE’s conformational cipher results show that the binary ciphertext generated in all tests was random.

### 4.2. Performance Experiment

To compare and evaluate the performance of the encryption/decryption time and reception rate, a processor environment that mimics an actual ECU must be constructed and simulated. However, the ECUs in each module that are responsible for specific functions in a real vehicle receive and process CAN messages owing to resource limitations. Therefore, in-vehicle infotainment (IVI) was used for encryption tests in an ECU module as part of a CAN. Currently, the maximum central processing unit (CPU) clock of a processor used in an IVI in most automobiles is 800 MHz. A Raspberry Pi was used for our experiments as it has a similar clock speed. Because the data frame of the CAN message can contain at maximum 8 bytes of data, it measures the time 10,000 times for the length of plain text from 2 bytes to 8 bytes of the meaningful minimum length and the encryption/decryption rate of the symmetric key algorithm. Additionally, the average was calculated to compare the encryption speed and decryption speed of FPE and the symmetric key algorithm.

The experimental results as Table 6 show that the AES-128 algorithm generates the block cipher fastest, followed by TDEA, and finally FPE_FF1. The symmetric key algorithms TDEA and AES-128 showed little difference in speed. FPE_FF1, which is an FPE algorithm, is a factor 15 to 50 slower than the basic AES-128 algorithm because rounds are performed several times based on AES-128. However, it is expected that the amount of data to be processed by each ECU module will increase owing to the development of automobile-related technologies in the future, and the specifications of ECUs for autonomous vehicles will be upgraded. Therefore, the same experiment was conducted with a faster CPU and assuming the specifications of ECUs to be used in future autonomous vehicles.

**Figure 7:**
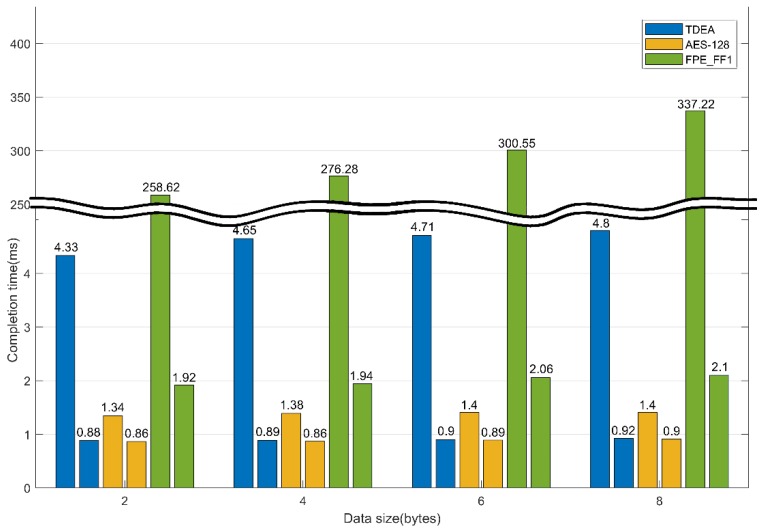
Encryption time comparison at 0.8 and 2.2 Ghz.

Table 7 and Figure 7 show the encryption speeds. The faster CPU allows the ECU to process more data in a given time. However, because TDEA generates 64 bits ciphertext, TDEA encrypts an unnecessary set of data when encrypting a message smaller than 64 bits, thus wasting CPU resources. AES-128 is used to generate 128 bits ciphertext, and data must be transmitted through at least two messages so that the receiver can normally decrypt and check the message. Therefore, if the FPE algorithm is used, it is possible to use resources efficiently by encrypting the data with a single message. A message is encrypted with 8 bytes, hexadecimally, according to the standard of the CAN. FPE is more efficient compared to other symmetric key algorithms because it can transmit only one message. In addition, it is considered that the algorithm speed of the FPE can be applied to the CAN when the ECU with an improved performance is developed through further research and development.

When receiving and processing a message on the CAN, AES-128 must be used to encrypt two messages, so we checked the reception ratio of messages transmitted from the CAN bus to each ECU. As an attacker increased the delay time of the message by randomly sending a higher priority message, an experiment was conducted to check whether a normal message could be received.

**Figure 8:**
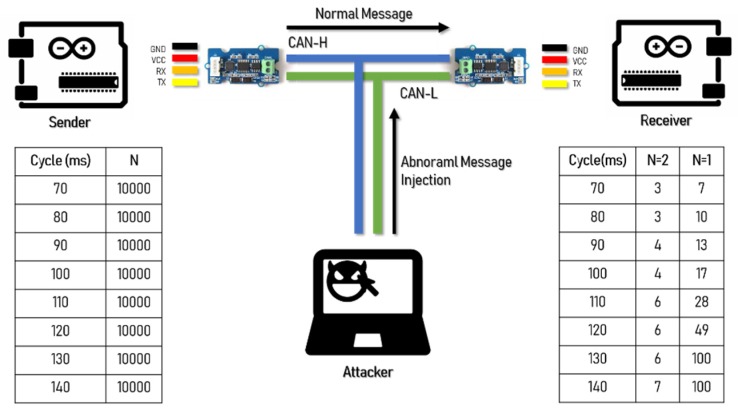
Experiment environment of the transmission rate controller area network (CAN) message.

We created an experimental environment with a Serial to CAN module (Logan) to generate and transmit a CAN message from one ECU. Another ECU then receives and confirms the message. The experimental environment is shown in Figure 8 [20].

**Figure 9:**
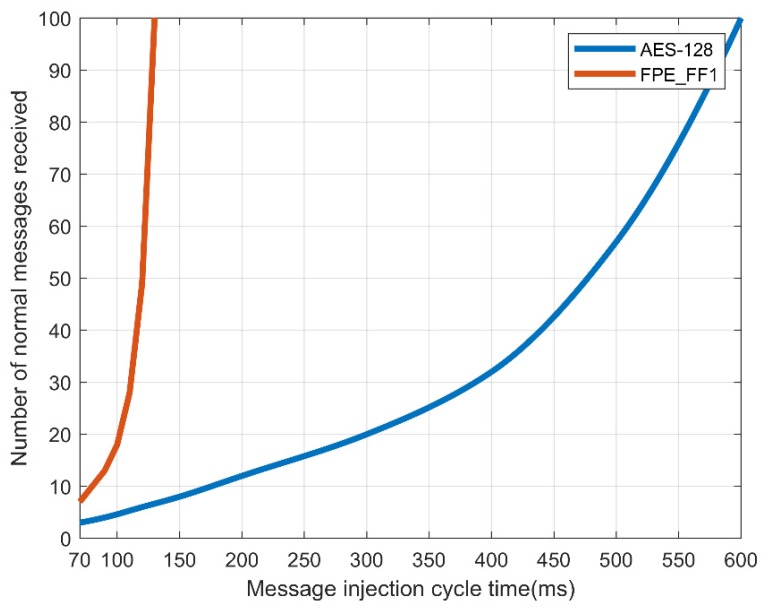
Success rate of message reception by number of controller area network (CAN) messages in a normal message period.

**Figure 10:**
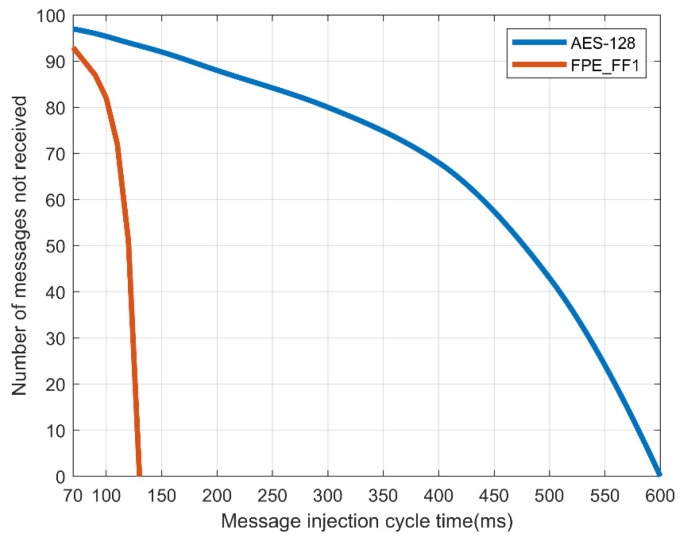
Loss rate of message reception by number of controller area network (CAN) messages in a normal message period.

When the normal message period is 100 ms, the attack speed of the CAN is delayed as in a denial-of-service attack as a function of the message injection rate. When the transmission speed of CAN was delayed, in the case of AES-128, to receive all N = 2 packets normally, a transmission speed of no less than 600 ms was required. In the case of the FPE, only the message of N = 1 was received in the same manner as the existing CAN message, requiring a transmission speed of no less than 130 ms. This means that, when encrypting and transmitting a CAN message, there is a delay in normal processing with AES-128, so there is a limit in processing the message normally compared to FPE. Figure 9 and Figure 10 show the message transfer rate.

In addition, we measured the normal reception rate for 100 messages with a normal message period ranging from 150 to 500 ms. The experimental results are shown in Table 8.

**Figure 11:**
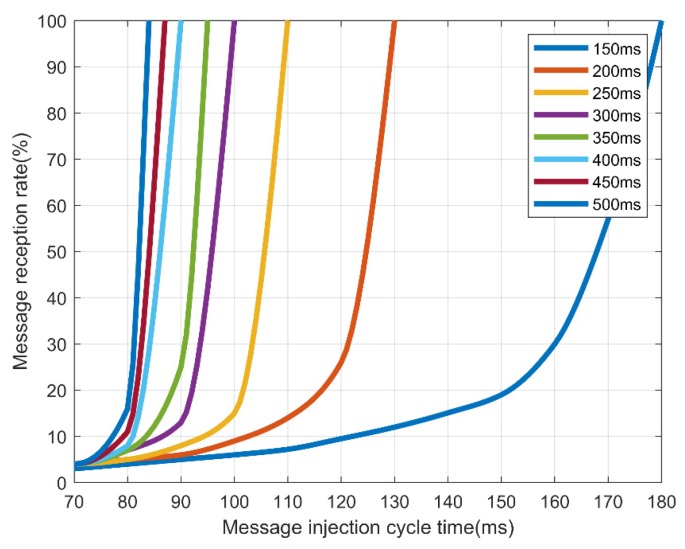
Reception rate by the message injection rate and message transmission cycle in AES-128.

**Figure 12:**
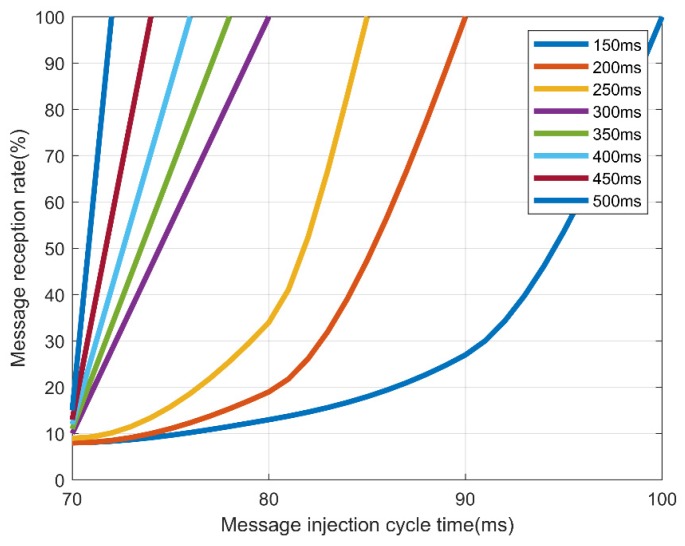
Reception rate by the message injection rate and message transmission cycle in format-preserving encryption (FPE)-FF1.

Table 9 and Figure 11 show that for AES-128, normal messages could be received when an injected message lasted at least 180 ms. However, Table 10 and Figure 12 in the case of FPE, normal messages could be received in every cycle, even if an abnormal message lasting at least 100 ms was injected. Therefore, FPE shows a better message reception rate than AES-128.

## 5. Discussion

Applying the proposed scheme to a real CAN requires a sufficient processing speed. There are differences in the CAN buses between each manufacturer and model, and a CAN bus provides different functions. According to the manufacturer A vehicle, body-related modules, e.g., B-CAN, to which ECU modules related to the external body frame, and those for controlling information necessary for actual vehicle operation, are connected. Additionally, with difficultly, the three CAN buses are physically divided, such as C-CAN and M-CAN.

**Figure 13:**
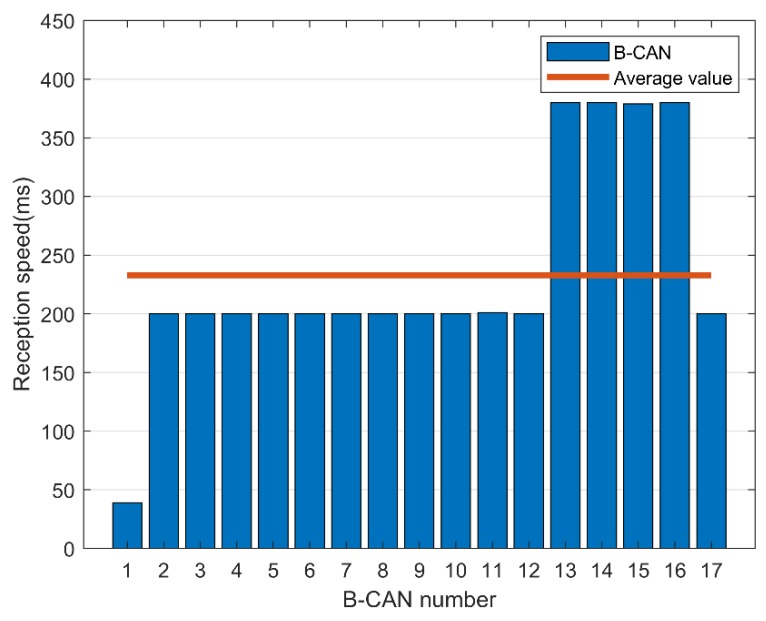
Message reception speed in B-controller area network (CAN).

**Figure 14:**
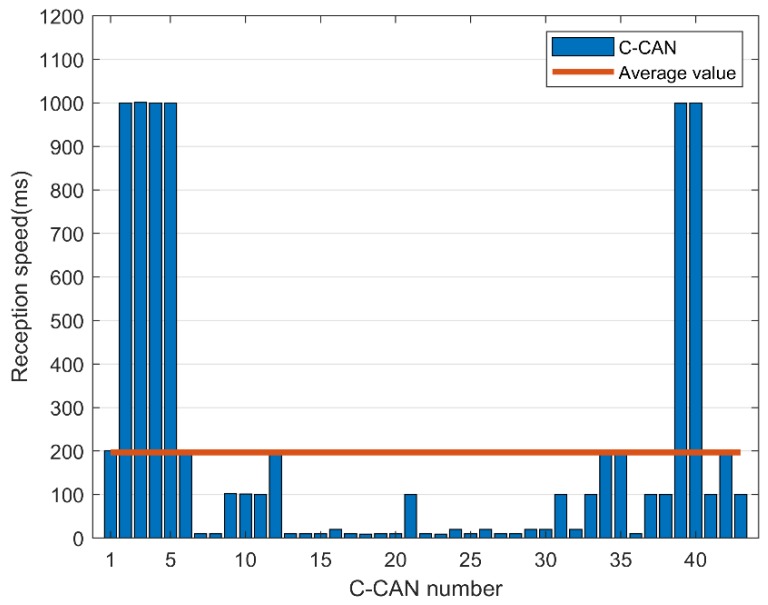
Message reception speed in C-controller area network (CAN).

There are messages that require a fast response time, such as those related to pressing the accelerator or braking, while other messages require a relatively slow response speed, such as those associated with opening or closing a door and controlling the volume. The average speed required to collect and analyze CAN messages is shown in Figure 13 and Figure 14.

In B-CAN, a message was continuously transmitted with a cycle of 200 ms on average, and in C-CAN, a message was continuously transmitted with a cycle of 100 ms on average. Therefore, a low response time of 100 ms is required to properly process the CAN message, and a fast encryption speed is required to accommodate this. We can apply the proposed FPE scheme using BeagleBone Black, which supports OpenSSL encryption acceleration [21].

The scheme ensures that the CAN frame is encrypted to ensure data confidentiality and only the user with the pre-shared key and Tn can encrypt the data, thus confirming that a CAN message is encrypted by the correct user. In addition, a fuzzing attack that injects arbitrary messages or a message insertion attack that induces malicious actions can be prevented. However, this scheme is still vulnerable to message authentication and denial-of-service attacks. In the case of secure hash algorithm (SHA)-based HMAC, the encryption speed is independent of the length of the plaintext. Therefore, a security element such as a hash function or HMAC can be used to authenticate the message. If you only receive the correct message using HMAC and try to decrypt it, you can prevent denial-of-service attacks.

## 6. Conclusions

We have proposed a scheme to encrypt CAN messages using FPE, which preserves the shape of the data frame and improves security. With this scheme, it is possible to guarantee data confidentiality without changing the CAN data frame. Furthermore, it is difficult to deduce plaintext from the ciphertext, regardless of the length of the plaintext, owing to the periodically changed tweak value when encrypting small amounts of data in a CAN message. Therefore, the data can be kept confidential without adding new elements to a CAN. To determine whether the proposed scheme is applicable to a real ECU in a CAN environment, we measured the encryption and decryption rates with various algorithms using a clock speed that is similar to that used in IVI, which is one of the best performing ECUs. Consequently, the processing speed was only about two times greater compared with the block cipher AES-128. In the case of the AES-128 algorithm, which has a relatively high speed, the result of receiving a success rate of the message by the denial-of-service attack is low because two messages must be used to transmit ciphertext. However, with FPE, CAN data frames can be transmitted without change, which increases the success rate of receiving messages and prevents denial of service (DoS). Therefore, this scheme can be used in an existing CAN environment and is more stable in CAN message processing. It is expected that the available processing speed will increase as high-speed ECUs are developed.

## Figures and Tables

**Table 1:** Description of the controller area network (CAN) data frame [4].

Field	Size	Feature
SOF	1 bit	The start bit of a frame indicates the beginning of a message, and the CAN bus is used to synchronize the message.
Identifier	11 bits	This determines the priority of a message. A low number means low priority, whereas a high number means high priority.
RTR	1 bit	When a node requests information from other remote node, all nodes receive the request and a response. The specific node processes the request based on the identifier and sends the response.
IDE	1 bit	Identifier extension bits indicate whether standard CAN frames are transmitted without extension or not.
R	1 bit	Reserved for later use.
DLC	4 bits	The number of bytes in the CAN data to be transferred.
Data	0–64 bits	This contains the actual data to be transmitted.
CRC	16 bits	16 bits checksum used for data error detection.
ACK	1 bit	Used to prove data integrity with 2 bits, and normal message reception uses the first bit.
EOF	7 bits	Identifies the end of a frame.

**Table 2:** Comparison of controller area network (CAN) message protection schemes.

Ref.	Encryption	Message Authentication	Security Elements
TESLA [11]	No	Yes	HMAC
CaCAN [12]	No	Yes	HMAC, Monitoring Node
CAN-Auth [13]	No	Yes	HMAC, Counter
LiBra-CAN [14]	No	Yes	M-MAC
LCAP [15]	Yes	Yes	SHA256, RC4
Vector [16]	Yes	No	ID Counter, AES

**Table 3:** Brief description of acronyms.

Notation	Description
*na*, *nb*	Random number
*K*	Pre-shared master keys
$T_{s}$	Tweak seed
$T_{n}$	*n*th tweak
*SK*	Session key
*H()*	Hash algorithm
*data*	Data field of CAN message
*fpeE()*	Encryption by FPE
*fpeD()*	Decryption by FPE
*C*	FPE-encrypted ciphertext

**Table 4:** Brief description of acronyms.

Notation	Description
*CANdata*	The data field value of the CAN message
*[x]^s^*	Change integer x to s byte string
*BC_K_(X)*	Encrypt X with the specified block cipher using key *K*
*BTN(X)*	Change bit string X to decimal integer
*NTR(X)*	Change number string X to hexadecimal integer
*ST_m_(X)*	Change positive integers X less than 16 m to hexadecimal digits of length m
*BEF(X)*	Encrypt the bit string X using the block cipher specified in CBC mode

**Table 5:** The *p*-value of the National Institute of Standards and Technology (NIST) 800-32 test results.

No.	Test Name	*p*-Value	
		FPE_FF1	AES
1	Frequency (Monobit) Test	0.496851469	0.502100334
2	Frequency (Block) Test	0.493339132	0.495865406
3	Runs Test	0.497479526	0.497193269
4	Test for the Longest Run of Ones in a Block Test	0.497265955	0.493814458
5	Binary Matrix Rank Test	0.296950751	0.292587342
6	Discrete Fourier Transform (Spectral) Test	0.490098975	0.484243809
7	Non-overlapping Template Matching Test	0.445934938	0.561494965
8	Overlapping Template Matching Test	0.019208297	0.019028279
9	Maurer’s Universal Statistical Test	0.958077761	0.957425343
10	Linear Complexity Test	0.610700126	0.606309826
11	Serial Test 1	0.503997652	0.501137345
	Serial Test 2	0.514842887	0.497810223
12	Approximate Entropy Test	0.484660249	0.479962347
13	Cumulative Sums Test	0.524336603	0.526251805
14	Random Excursions Test	0.584221171	0.581522582
15	Random Excursions Variant Test	0.639011415	0.644466544

**Table 6:** Encryption/decryption time comparison using Raspberry Pi (1 Ghz).

	Algorithm	TDEA		AES-128		FPE_FF1	
Data Size		Encryption	Decryption	Encryption	Decryption	Encryption	Decryption
2 bytes		2.8 ms	9.7 ms	2.6 ms	3.6 ms	160.3 ms	164.4 ms
4 bytes		3.7 ms	11.2 ms	2.7 ms	3.9 ms	220.4 ms	229.3 ms
6 bytes		5.5 ms	12.3 ms	2.9 ms	4.6 ms	255.0 ms	262.2 ms
8 bytes		8.8 ms	12.9 ms	3.1 ms	5.5 ms	289.6 ms	301.9 ms

**Table 7:** Encryption time with 0.8 and 2.2 GHz CPUs.

Algorithm	TDEA		AES-128		FPE_FF1	
CPU Clock (GHz)	**0.8**	**2.2**	**0.8**	**2.2**	**0.8**	**2.2**
2 bytes	4.335 ms	0.882 ms	1.347 ms	0.867 ms	258.626 ms	1.92 ms
4 bytes	4.65 ms	0.89 ms	1.3898 ms	0.869 ms	276.284 ms	1.941 ms
6 bytes	4.711 ms	0.9 ms	1.4052 ms	0.892 ms	300.599 ms	2.06 ms
8 bytes	4.801 ms	0.92 ms	1.4055 ms	0.908 ms	337.288 ms	2.103 ms
Average	4.624 ms	0.898 ms	1.3868 ms	0.884 ms	293.174 ms	2.006 ms
Throughput (sec/byte)	8.65	0.1796	2.88	0.1768	58.63	0.4012

**Table 8:** Controller area network (CAN) message reception rate.

Message Injection Cycle Time	AES-128 (N = 2)		FPE_FF1 (N = 1)	
	Normal	Abnormal	Normal	Abnormal
70 ms	3%	97%	7%	93%
80 ms	3%	97%	10%	90%
90 ms	4%	96%	13%	87%
100 ms	4%	96%	18%	82%
110 ms	6%	94%	28%	72%
120 ms	6%	94%	49%	51%
130 ms	6%	94%	100%	0%
140 ms	7%	93%		
150 ms	7%	93%		
200 ms	12%	88%		
300 ms	20%	80%		
400 ms	32%	68%		
500 ms	57%	43%		
600 ms	100%	0%		

**Table 9:** Controller area network (CAN) message reception rate with AES-128.

Cycle	70	80	90	100	110	120	130	140	150	160	170	180
150 ms	3%	4%	5%	6%	7%	10%	12%	16%	19%	30%	57%	100%
200 ms	4%	5%	6%	9%	14%	26%	100%					
250 ms	4%	5%	8%	15%	100%							
300 ms	4%	7%	13%	100%								
350 ms	4%	7%	25%	100%								
400 ms	4%	8%	100%									
450 ms	4%	11%	100%									
500 ms	4%	16%	100%									

**Table 10:** Controller area network (CAN) message reception rate with format-preserving encryption (FPE)-FF1.

Cycle Time	70	80	90	100
150	8%	13%	27%	100%
200	8%	19%	100%	
250	9%	34%	100%	
300	10%	100%		
350	11%	100%		
400	12%	100%		
450	13%	100%		
500	15%	100%		
